# Identification, expression and characterization of the recombinant Sol g 4.1 protein from the venom of the tropical fire ant *Solenopsis geminata*

**DOI:** 10.1186/s40409-018-0159-6

**Published:** 2018-08-29

**Authors:** Hathairat Srisong, Sophida Sukprasert, Sompong Klaynongsruang, Jureerut Daduang, Sakda Daduang

**Affiliations:** 1 Protein and Proteomics Research Center for Commercial and Industrial Purposes (ProCCI), Department of Biochemistry, Faculty of Science, Khon Kaen University, Khon Kaen, 40002 Thailand; 2 Division of Integrative Medicine, Chulabhorn International College of Medicine, Thammasat University (Rangsit Campus), Pathum Thani, 12120 Thailand; 3 Department of Clinical Chemistry, Faculty of Associated Medical Sciences, Khon Kaen University, Khon Kaen, 40002 Thailand; 4 Division of Pharmacognosy and Toxicology, Faculty of Pharmaceutical Sciences, Khon Kaen University, Khon Kaen, 40002 Thailand

**Keywords:** Fire ant, Sol g 4.1 protein, Allergen, Venom protein, Stinging ant

## Abstract

**Background:**

Fire ant venom is a complex mixture consisting of basic piperidine alkaloids, various biologically active peptides and protein components, including a variety of major allergenic proteins. Tropical fire ant *Solenopsis geminata* is an important stinging ant species that causes anaphylaxis and serious medical problems. Although the biological activities of allergenic venom proteins that are unique to ant venom, particularly *Solenopsis* 2 and 4, are still unknown, these proteins are believed to play important roles in mediating the effects of the piperidine derivatives in the venom.

**Methods:**

In the present study, the cDNA cloning, sequencing and three-dimensional structure of Sol g 4.1 venom protein are described. The recombinant Sol g 4.1 protein (rSol g 4.1) was produced in *E. coli*, and its possible function as a hydrophobic binding protein was characterized by paralyzing crickets using the 50% piperidine dose (PD_50_). Moreover, an antiserum was produced in mice to determine the allergenic properties of Sol g 4.1, and the antiserum was capable of binding to Sol g 4.1, as determined by Western blotting.

**Results:**

The molecular weight of Sol g 4.1 protein is 16 kDa, as determined by SDS-PAGE. The complete cDNA is 414 bp in length and contains a leader sequence of 19 amino acids. The protein consists of six cysteines that presumably form three disulfide bonds, based on a predicted three-dimensional model, creating the interior hydrophobic pocket and stabilizing the structure. The rSol g 4.1 protein was expressed in inclusion bodies, as determined by SDS-PAGE. Dialysis techniques were used to refold the recombinant protein into the native form. Its secondary structure, which primarily consists of α-helices, was confirmed by circular dichroism analysis, and the three-dimensional model was also verified. The results of allergenic analysis performed on mice showed that the obtained protein was predicted to be allergenically active. Moreover, we report on the possible role of the Sol g 4.1 venom protein, which significantly reduced the PD_50_ from 0.027 to 0.013% in paralyzed crickets via synergistic effects after interactions with piperidine alkaloids.

**Conclusions:**

The primary structure of Sol g 4.1 showed high similarity to that of venom proteins in the *Solenopsis* 2 and 4 family. Those proteins are life-threatening and produce IgE-mediated anaphylactic reactions in allergic individuals. The possible function of this protein is the binding of the interior hydrophobic pockets with piperidine alkaloids, as determined by the analysis of the structural model and PD_50_ test.

**Electronic supplementary material:**

The online version of this article (10.1186/s40409-018-0159-6) contains supplementary material, which is available to authorized users.

## Background

Fire ants of the genus *Solenopsis*, which originally came from South and Central America, are distributed in tropical regions across the globe [1–4]. According to international reports, ant hypersensitivity is currently one of major causes of severe systemic reactions or anaphylaxis [5, 6]. The majority of fire ant venom consists of 90–95% basic piperidine alkaloids, which are produced in the venom glands, stored in the poison sac and dispensed through the stinging apparatus [7, 8]. The alkaloids are mainly hydrophobic piperidines composed of different combinations of the same 2,6-dialkylpiperidines [9]. These alkaloids function primarily in defense, colony hygiene, and food procurement and have physiological functions such as histamine-releasing, antibacterial, antifungal, insecticidal, phytotoxic and hemolytic properties [10–13]. The alkaloid causes the formation of the characteristic pustule, burning sensation and sterile necrotic lesions at the site of envenomation [14].

The small aqueous phase of venom contains four major proteins that are responsible for the allergenic activity [15]. A single fire ant sting contains only 10–100 ng of protein and can cause specific IgE antibody production [1]. Four allergenic proteins have been isolated from *Solenopsis invicta* (*S. invicta*) venom and characterized [15, 16]. Sol i 1 is a phospholipase A_1_ and belongs to the lipoprotein lipase family; it is similar to a version found in wasp venom [17]. Sol i 3 is a member of an antigen 5 protein family with an unknown biological function [18]. Sol i 2 and Sol i 4 are unique to ant venoms and do not seem to be homologous to any bee or vespid venom proteins [16]; their biological functions are still unknown.

Sol i 2 makes a covalent bond to form a homodimer. Each molecule consists of seven Cys residues: six cysteines form three intramolecular disulfide bonds that stabilize the structure, and the seventh cysteine (Cys22) links two monomers by a disulfide bond [15, 19, 20]. Proteins similar to Sol i 2 are found in the venom of other *Solenopsis* species, including *Solenopsis geminata* (Sol g 2), *Solenopsis richteri* (Sol r 2), *Solenopsis saevissima* (Sol s 2), and *Solenopsis xyloni* (Sol × 2) [3, 21]. Sol i 4 is related to Sol i 2, sharing 37% sequence identity, and is 118 amino acids long. It lacks the dimerizing cysteine and carbohydrate and is present in venom as a monomer [22]. Sol i 4 comprises 8–10% of the venom protein and is the most basic protein component [1, 22]. Proteins similar to Sol i 4 have been identified in the venom of *S. geminata* species (Sol g 4). Sol g 4 has two isoforms that are 97% identical, and other isoforms are 90% identical to Sol i 4. Venom toxicity is expected to be caused by solenopsins and methyl-, alkyl- or alkenyl-substituted piperidines [23]. The venom has cytotoxic, insecticidal, antibiotic and antimicrobial properties as well [11, 24].

The morphology and venom composition of *S. invicta* are similar to those of *Solenopsis* species in tropical areas, including *S. geminata* [1, 6]. The venom of the tropical fire ant *S. geminata* produces anaphylaxis and serious medical problems in Taiwan, Indonesia and many Asian islands and in Thailand [5]. *S. geminata* is widely distributed throughout all areas in Thailand, and these ants are commonly found in houses and fields [25, 26]. Major components are piperidine alkaloids [1, 27]. Although other components, including unidentified soluble insect proteins, comprise a small proportion of venom, they play important roles in venom action. Therefore, in this study, we identified and sequenced Sol g 4.1, a major protein component of *S. geminata* venom, using a comparative study. We produced the recombinant Sol g 4.1 protein in *E. coli* and characterized it to better understand its properties, including allergenic properties, and possible functions.

## Methods

### Fire ant venom collection and gland extraction

*Solenopsis geminata* is normally found throughout Thailand. Adult *S. geminata* workers were collected from suburban areas of Khon Kaen City, Khon Kaen Province, in the dry season from January to April 2013. Venom from the tips of the stingers was collected with capillary tubes under a magnifying glass and stored at − 20 °C in PBS until use. All bottom insect parts were chopped for a single large-scale extraction, with a homogenate: PBS ratio of 1:200 *w*/*v*. The extract was centrifuged at 10,000 rpm for 10 min, and the supernatant was separated and stored at − 80 °C until use. The protein contents were quantitatively determined by the Bradford method [28] using bovine serum albumin as the standard.

### Isolation of mRNA and first strand cDNA synthesis

Approximately 1 g of whole *S. geminata* bodies was frozen in liquid nitrogen and homogenized. RNA was isolated using TRIzol® reagent (Invitrogen, Life Technologies, USA). Briefly, the homogenized ants were dissolved in 1 mL of TRIzol® reagent and centrifuged at 12,000×g for 10 min at 4 °C. The homogenized sample was incubated for 5 min at room temperature and then 0.2 mL of chloroform was added. The sample was centrifuged at 12,000×g for 15 min at 4 °C. The aqueous phase was incubated with 0.5 mL of isopropanol at room temperature for 10 min and centrifuged at 12,000×g for 10 min at 4 °C. The pellet was washed with 1 mL of 75% ethanol. The sample was mixed by vortexing and centrifuged at 7,500×g for 5 min at 4 °C. The supernatant was discarded. The RNA pellet was dried for 10–20 min and re-suspended in diethyl pyrocarbonate- (DEPC) treated water by passing the solution through a pipette tip a few times. The solution was stored at − 80 °C until use. RT-PCR was performed to synthesize the first-strand cDNAs with oligo (dT)_18_ primer and RevertAid First strand cDNA synthesis kit (Thermo Scientific, USA), as described in the instruction manual.

### Protein identification by liquid chromatography coupled with mass spectrometry (LC-MS/MS)

In-gel digestion and mass spectrometry techniques were performed using the methods described by Sukprasert et al. [26]. Briefly, the endogenous Sol g 4.1 protein and the purified recombinant protein were separated by native-PAGE and SDS-PAGE (sodium dodecyl sulfate polyacrylamide gel electrophoresis), respectively. Both natural and recombinant Sol g 4. 1 proteins were excised, washed and digested with 20 ng/spot of modified trypsin (Promega, USA) in 50% acetonitrile/10 mM ammonium bicarbonate at 37 °C for 3 h. Peptides were extracted by washing the gel pieces three times with 200 μL of 50% acetonitrile/0.1% formic acid. The supernatant was dried at 37 °C for 3 h, dissolved in 0.1% (*v*/v) formic acid and stored at 30 °C until the mass spectrometry analysis.

The sample was then subjected to the Ultimate 3000 LC System (Dionex) coupled with an ESI-Ion trap MS (HCTultra PTM Discovery System, Bruker Daltonik). A database was generally searched to identify peptides identification using a local MASCOT server and the following search parameters: NCBI proteins and SwissProt for protein databases, a specified trypsin enzymatic cleavage with one possible missed cleavage, ±0.6 Da mass tolerances for MS and MS/MS, a peptide tolerance of ±0.5 Da, 1+, 2+, and 3+ ions, methionine oxidation as the variable modification, carbamidomethyl (C) as the fixed modification, and monoisotopic mass.

### Polymerase chain reaction amplification

A degenerate sense oligonucleotide primer was designed according to the sequence similarity of the conserved region of Solenopsis 4 venom proteins and the nucleotide sequences corresponding to peptide sequences obtained from previous studies [26]. The RACE procedures were performed using the RACE System (Invitrogen, Life Technologies, USA). The 3΄-RACE and 5΄-RACE reactions were performed with a gene-specific primer and common primers listed in Table 1. PCR was performed for 30 cycles: 30 s at 94 °C, 1 min at 58 °C, and 1 min at 72 °C. The final extension step was performed for 7 min. The DNA fragment was verified with a sense primer (Fsol4_Nco) and an antisense primer (Rsol4_Xho). All sequences were verified by sequencing dependently derived clones.

**Table 1:** List of primers used in PCR and RACE-PCR

Degenerate	
F2 5΄-AAWGTATRAAWACAVHAYC-3΄	
R3 5΄-CKTYTBYCAWTKATTRGTSCC-3΄	
RACE	
3RACE 5΄-CGCAGCTGATATTAAGG-3΄	
5RACE 5΄-GTCAATTCGAGCACACCC-3΄	
PCR	
FSol4_Nco 5΄-*CCATGG*CTGCTGATATTAAGGA-3΄	
RSol4_Xho 5΄-*CTCGAG*TCATTTTTTTTTGCCATAC-3΄	
Common	
Oligo (dT_18_) 5΄-GGCCACGCGTCGACTAGTACTTTTTTTTTTTTTTTTT-3΄	
AAP 5΄-GGC CAC GCG TCG ACT AGT ACG GGI IGG GII GGG IIG-3΄	

The PCR product of Sol g 4.1 lacking a leader sequence was ligated into the pGEM-T easy vector (Promega Inc., USA) and transformed into competent DH5α *E. coli* t cells (Invitrogen, USA). After transformation, positive colonies were screened by colony PCR using the conditions described above. The transformants were confirmed by extracting recombinant plasmids, digesting them with restriction enzymes and performing agarose gel electrophoresis. Moreover, the coding sequences of the recombinant plasmids were confirmed by First BASE Laboratory (Seri Kembangan, Selangor, Malaysia), which used the T7 promoter forward and T7 terminator reverse primers.

### Sequence analysis and structural modeling

The basic characterization of gene and protein sequences was performed using the NCBI database (http://www.ncbi.nlm.nih.gov/) and the basic local alignment search tool BLAST (https://blast.ncbi.nlm.nih.gov/). The molecular weight and isoelectric points were computed using the Compute pI/MW tool provided by ExPASy Bioinformatics (https://www.expasy.org/). The three-dimensional structure was modeled using the Swiss-Model System and the automated protein homology modeling server at ExPASy (Switzerland) [29]. The X-ray crystal structure of the venom allergen 2 (Sol i 2) monomer from *S. invicta* (PDB code: 2ygu) venom was used as a template for computational homology modeling. The three-dimensional models were visualized and compared using the UCSF Chimera program (http://www.cgl.ucsf.edu/chimera/). The stereochemical quality validation of model was performed by PROCHECK tool including Ramachandran plot.

### Expression of the rSol g 4.1 protein

The Sol g 4.1 gene was subcloned from the pGEM-T easy vector into the pET-32a expression vector (Invitrogen, UK). Briefly, the vectors were double digested with NcoI and XhoI restriction enzymes, and the Sol g 4.1 gene was ligated into the same restriction sites of the pET-32a expression vector. Recombinant plasmids were transformed into *E. coli* BL21 (DE3) pLysS competent cells (Promega, Malaysia). A single colony from a freshly streaked plate was picked, inoculated in LB (Sigma-Aldrich, USA) starter media containing 50 μg/mL ampicillin and incubated at 37 °C overnight with shaking until the culture was turbid but not saturated.

The 5-mL starter cultures were transferred into 500 mL of LB expression medium containing 100 μg/mL ampicillin and incubated at 37 °C until the cell density reached to OD_600_ of ~ 0.5. Afterwards, the temperature was reduced to 30 °C and the culture was induced with 0.4 mM IPTG. The induced cultures were grown for 8 h. Cell pellets were collected and washed with 10 mL of lysis buffer (20 mM sodium phosphate, pH 7.4, 100 mM NaCl, 1 mM DTT, and 0.1 mM PMSF) and disrupted by sonication on ice. After centrifugation at 15,000×g for 20 min at 4 °C, the supernatants were separated on 13% SDS-PAGE gels.

### Refolding and purification of the rSol g 4.1 protein

The rSol g 4.1 protein with the polyhistidine tag was detected as an insoluble protein; therefore, the induced cell pellets were sonicated with lysis buffer on ice, dissolved in 20 mL of buffer A (20 mM sodium phosphate pH 7.4, 8 M urea, and 1 mM DTT) and incubated with shaking for 3 h. After centrifugation at 15,000×g for 10 min at 4 °C, the rSol g 4.1 protein was refolded into the conformation with the correct intramolecular associations by dialysis against 50 volumes of buffer B (20 mM sodium phosphate buffer pH 7.4, 10% glycerol, 0.1 mM EDTA, 1 mM DTT, 100 mM NaCl, 0.1 mM PMSF) and in solutions with gradually reduced urea concentrations until the buffer was urea-free for 3 h at 4 °C in each buffer. Finally, the protein was dialyzed against buffer C (20 mM sodium phosphate buffer pH 7.4, 10% glycerol, 1 mM DTT, 100 mM NaCl, and 0.1 mM PMSF) overnight.

The rSol g 4.1 protein was purified using a His GraviTrap column (GE Healthcare, USA) according to the manufacturer’s instructions. Briefly, the column was equilibrated with 10 mL of binding buffer (20 mM sodium phosphate, 500 mM NaCl and 20 mM imidazole, pH 7.4). After the sample was loaded, the column was washed with 10 column volumes of binding buffer to remove the contaminating proteins and eluted with 5 mL of elution buffer (20 mM sodium phosphate, 300 mM NaCl and 300 mM imidazole, pH 7.4). Each eluted fraction was analyzed by 13% SDS-PAGE and dialyzed against 10 mM sodium phosphate buffer, pH 7.4.

The rSol g 4.1 protein was cleaved with enterokinase (Sigma-Aldrich, USA) to remove the tag from the protein, according to the manufacturer’s instructions. Enzyme aliquots of 0.1, 0.2, 0.5 or 1 U were mixed with reaction buffers and 1 mg of rSol g 4.1 protein, and all reactions were then incubated for 2, 4, 7 or 16 h at room temperature. Each reaction was analyzed by 13% SDS-PAGE. Finally, the rSol g 4.1 protein lacking the tag was separated using a His GraviTrap column.

### SDS-PAGE and western immunoblotting

One-dimensional SDS-PAGE was performed according to a standard method using a 13% (w/v) separating gel and a 4% (w/v) stacking gel. Phosphorylase B (97 kDa), bovine serum albumin (66 kDa), chicken ovalbumin (45 kDa), carbonic anhydrase (30 kDa), trypsin inhibitor (20 kDa) and α-lactalbumin (14.4 kDa) were used as standards. After samples were applied to the gel, the proteins were resolved at 150 V for 1 h. Gels were stained with Coomassie brilliant blue R-250 (CBB).

For blots of the IgE reactivity test, the gel was placed in a blotting apparatus after electrophoresis, and proteins were electro-transferred to a nitrocellulose membrane for 1 h. The membrane was incubated with blocking solution (5% nonfat dry milk in TBST buffer). It was also incubated with antiserum diluted in blocking solution for 1 h, washed three times with TBST with shaking, and incubated with a 1:50 dilution of alkaline phosphatase-conjugated rat anti-mouse IgE (SouthernBiotech, USA) with rocking. The membrane was washed three times with TBST and TBS and then developed with BCIP/NBT (GE Healthcare, Sweden). The membrane was rinsed with water to stop the color development and allowed to dry. For blotting to confirm the size of the rSol g 4.1 protein, we used a 1:1,000 dilution of an anti-His tag antibody (Sigma-Aldrich, USA) as the primary antibody, and the protein was detected by incubating the membrane with a 1:8,000 dilution of an alkaline phosphatase-conjugated goat anti-mouse IgG (Sigma-Aldrich, USA).

### Polyclonal antibody production

The method reported by Dearman et al. [30] to produce antibodies in mice was applied here to study antiserum production in the sera of BALB/c strain mice. Crude venom proteins were separated by native-PAGE, and the band of Sol g 4.1 protein is proposed to be 16 kDa, as reported by Sukprasert et al. [26]. The band at this size was excised from the gel and frozen at − 70 °C. The gel was dried by lyophilization and then ground into a fine powder. The powder was rehydrated in 1–2 mL of PBS buffer (137 mM NaCl, 2 mM KH_2_PO_4_, 2.7 mM KCl and 10 mM Na_2_HPO_4_, pH 7.4). This protein suspension was mixed with an equal volume of Freund’s complete adjuvant (Sigma-Aldrich, USA) for emulsification. Mice were subcutaneously immunized with approximately 100 μL of emulsion. After 10 days, they were again boosted with protein and Freund’s incomplete adjuvant and injected 2–3 times every 10 days. Three days after every injection, blood was collected from the retro-orbital plexus using a 100-μL micropipette coated with 1 U/mL heparin [31]. The blood was stored at 4 °C after collection. The serum was collected by centrifugation at 10,000×g for 10 min, and the supernatant containing the antiserum was pooled. The titer and specificity of the antiserum was determined by ELISA and Western blotting techniques. An acrylamide gel fragment lacking protein was used as a control.

### Circular dichroism (CD) measurements

Estimations of the secondary structure were performed using CD with a 1 mg/mL solution in a quartz cell cuvette with a path length of 0.5 cm, a scanning rate of 100 nm min^− 1^ and a range of 190–260 nm on a Jasco J-815 CD Spectrometer (JASCO, Japan) at the Faculty of Science, Khon Kaen University. Excitation and emission spectra were recorded using a slit width of 5 nm, and absorption spectra were measured using an Agilent HP 8453 spectrophotometer. The CD spectra were analyzed to compare the secondary structures of the proteins using Spectra Manager II software. The CD spectra of refolded and non-refolded rSol g 4.1 proteins without the tag were compared. The non-refolded rSol g 4.1 protein was solubilized with 8 M urea and the refolded rSol g 4.1 protein was solubilized in 0.1 mM sodium phosphate buffer, pH 7.4, and CD spectra were recorded.

### Paralytic dose 50 (PD_50_) assay with piperidine derivatives

The PD_50_ test was used to determine possible functions of the refolded rSol g 4.1 protein lacking the tag, which may affect the interaction with piperidine alkaloids in paralyzed crickets (*Gryllus* sp.). A cricket body weight of 0.35 ± 1 g was used. The PD_50_ was defined as the concentration of piperidines (Sigma-Aldrich, USA) that paralyzed 50% of injected crickets; crickets that could not turn over from the dorsal, upright position were considered paralyzed. We designed experiments using three groups: one – injection with piperidine (2-methylpiperidine, C_6_H_13_N) alone, two – injection with the rSol g 4.1 protein alone, and three – injection with both piperidine and the rSol g 4.1 protein.

First, various concentrations of piperidine were mixed with PBS, pH 7.4, quantified, and then injected into the cricket abdomen. After 10 min, paralyzed crickets were counted and analyzed for PD_50_P1 [32]. Second, various concentrations of the rSol g 4.1 protein concentrations were injected alone, as described above. Finally, the optimal concentrations of rSol g 4.1 proteins that did not paralyze crickets were mixed with various piperidine concentrations. The PD_50_ values for the mixtures in paralyzed crickets were recorded and determined as PD_50_P2. All concentration tests used six crickets and were performed in triplicate. For the statistical analysis, the results are presented as means ± SEM (standard errors of the mean). According to reports adhering to the central limit theorem [33, 34], the sample data exhibited an approximately normal distribution and were subjected to unpaired t-test analysis.

## Results

### Full-length Sol g 4.1 protein

**Fig. 1:**
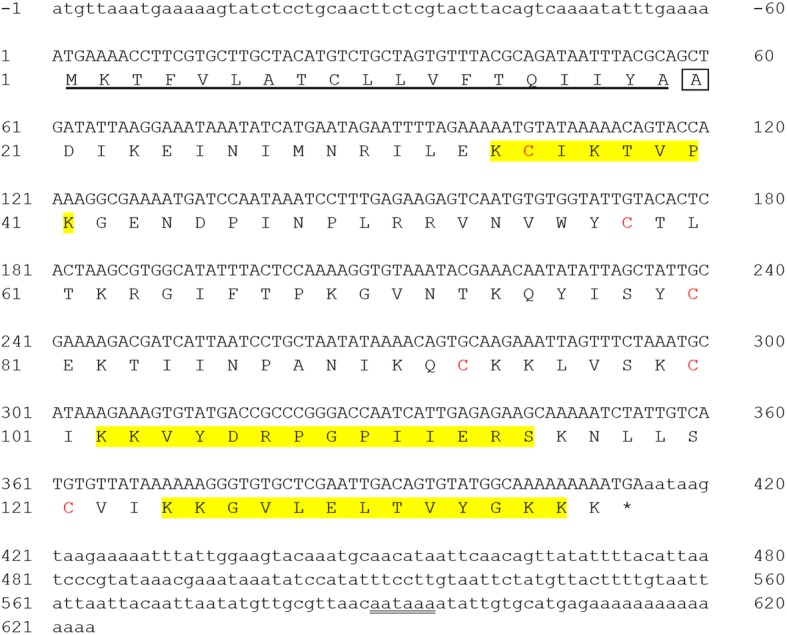
Full-length DNA sequence and translation of the region encoding the Sol g 4.1 protein. Yellow-shaded areas were verified by LC-MS/MS of a partial amino acid sequence. The leader sequence is underlined. The 5΄ and 3΄UTRs are indicated by small letters, and the poly (A) tail initiation signal is double underlined. The boxed residue was determined by automated Edman degradation sequencing. The red letters represent cysteines residues, and the termination codon is indicated by *

We utilized RT-PCR, PCR and standard cloning techniques to obtain the complete cDNA sequence of the *S. geminata* venom allergen Sol g 4.1. The middle section of the cDNA was cloned using degenerate primers (Table 1). PCR products were cloned, sequenced and analyzed. The sequence was used to choose exact primers for 3΄ and 5΄-RACE, as shown in Table 1. Amplification of the 3΄-fragments was performed using both oligo dT primers and the primer 3RACE. The 5΄-fragments were obtained with a matched known sequence from 3΄-RACE results using 5RACE and AAP primers. All sequences resulted from positive clones that were merged and identified. The full-length nucleotide sequence from 5΄UTR through the poly-A tail (3΄UTR) and the deduced amino acid sequence are shown in Fig. 1.

The complete coding sequence of the cDNA is 414 nucleotides to the stop codon, corresponding to 137 amino acids, including six cysteine residues after deduction, which are related to other published Solenopsis 4 venom proteins (Sol i 4.01, Sol i 4.02, and Sol i 4q) [35–37]. The signal sequence was analyzed using the Signal P program and identified 57 bp encoding 19 amino acids. The primary sequence of the deduced mature Sol g 4.1 protein contains 118 amino acid residues and starts with alanine (A), as confirmed by automated Edman degradation sequencing (data not shown).

**Fig. 2:**
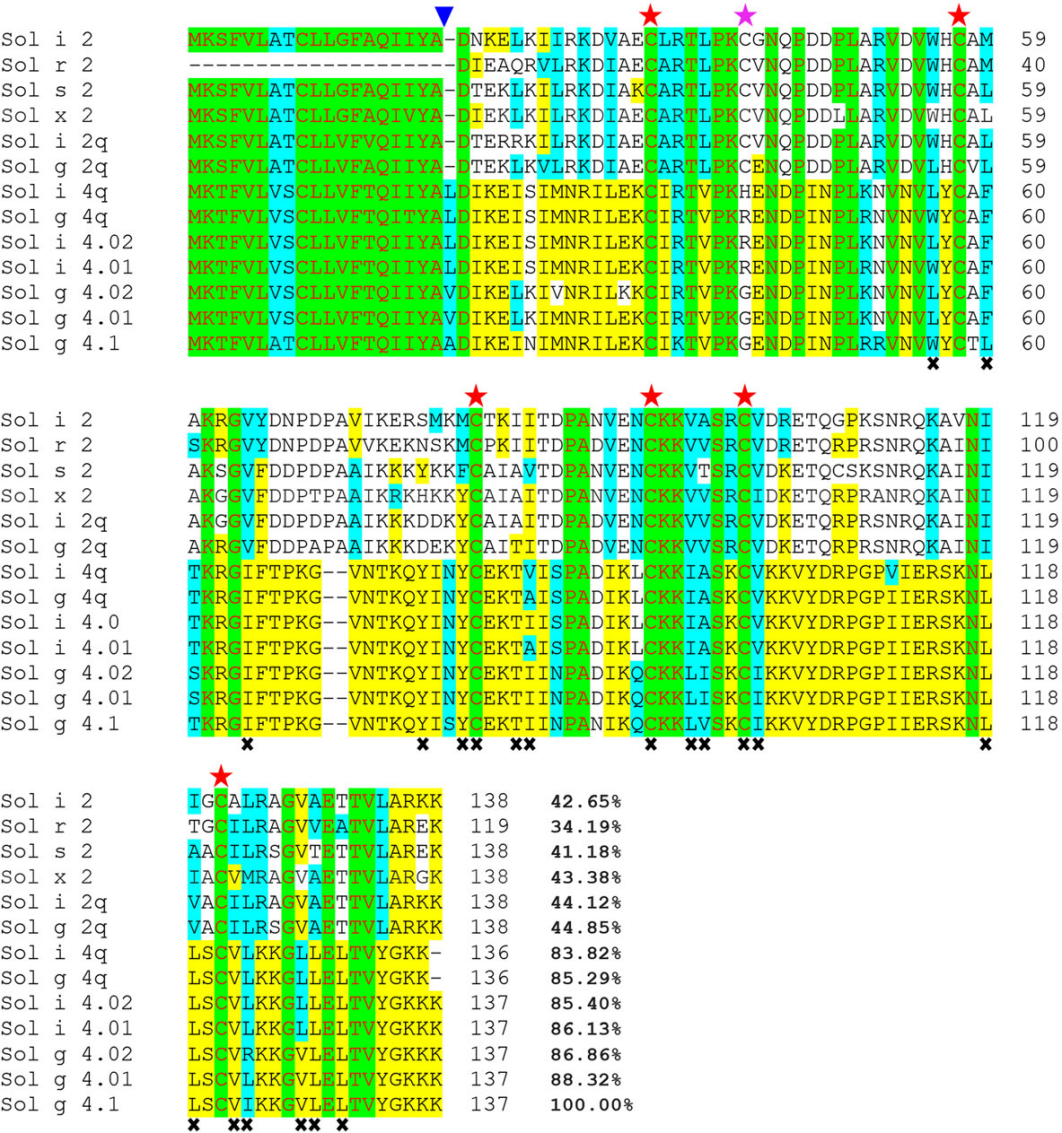
Alignment of the deduced amino acid sequences of the Sol g 4.1 protein with other Solenopsis 2 and 4 venom proteins from *S. invicta*, *S. geminata*, *S. saevissima*, *S. xyloni* and *S. richteri*: conserved (red letters, green region), identical (yellow region), and groups of similar (turquois region) or non-similar (black letters, no color region) residues are shown. The end of the signal sequence is indicated by a blue triangle (). Alignment of the six cysteines (red stars) between all Solenopsis 2 and 4 genes and the alignment of the seventh cysteine in the Sol 2 genes (pink star). Residues lining the interior face of the Sol g 4.1 protein are indicated by **x**. The sequences were submitted to GenBank with the following accession numbers: Solenopsis 2 proteins: P35775 for Sol i 2, P35776 for Sol r 2, ABC58726 for Sol s 2, ALM98859 for Sol × 2, AAY32928 for Sol i 2q and AAY32926 for Sol g 2q; and Solenopsis 4 proteins: AAC97369 for Sol i 4.01, AAC97370 for Sol i 4.02, AAF65312 for Sol g 4.01, AAF65313 for Sol g 4.02, AAY32927 for Sol g 4q and AAY32929 for Sol i 4q

Although the leucines (L) and valines (V) observed in Solenopsis 4 venom proteins are substituted with A residues in the Sol g 4.1 protein, these amino acids are classified into hydrophobic groups and followed by DIKE sequences that were all highly conserved, as shown in Fig. 2. The protein was rich in the amino acids K, N and P, with a theoretical isoelectric point of 9.87 and a predicted molecular weight of 13,527.50 Da. GenBank Blastx searches revealed that the Sol g 4.1 protein closely resembles a member of the unique Solenopsis 2 and 4 venom proteins, whose biological functions remain unknown.

### Comparison to Solenopsis 2 and 4

The alignment of the amino acid sequences of the Sol g 4.1 protein with the published Solenopsis 2 and 4 venom protein sequences from *Solenopsis* species is shown in Fig. 2. The comparison of these sequences showed that all six cysteines were conserved within Solenopsis 4 venom proteins, and all seven cysteines (including the six Solenopsis 4 cysteine positions) were conserved within the Solenopsis 2 venom proteins. The Sol g 4.1 venom protein shares 88.3% and 86.9% amino acid identity with the Sol g 4.01 and 4.02 allergens (GenBank ID: AAF65312 and GenBank ID: AAF65313), respectively; therefore, we designated this venom protein the Sol g 4.1 protein to differentiate between these proteins. The protein showed similarity to Sol i 4.01 and Sol i 4.02 (GenBank ID: AAC97369 and GenBank ID: AAC97370, respectively) (both 85%) [22, 36]. The identity among all sequenced Solenopsis 4 proteins ranged from 83.8 to 88.3%, illustrating that the Solenopsis 4 proteins are rarely diverse and on average exhibit 86.0% identity among all Solenopsis 4 venom proteins. These sequences are highly conserved across species but are still poorly understood. Only 28 of the 118 mature amino acid sequences closely matched Solenopsis 2 and 4 venom proteins, in contrast to other published reports. Interestingly, the signal peptides of both groups are highly conserved and contain the greatest number of hydrophobic amino acid groups.

The *Solenopsis* venom proteins were used to construct a phylogenetic tree and analyzed using MEGA6 software [38] to confirm these results (Additional file 1). The major finding of this analysis is the conservation of six cysteines among all Solenopsis 2 and 4 venom proteins, but the seventh cysteine was only present in group 2; it forms a disulfide bond identical to that in other molecules [39]. Interestingly, Sol g 4q (GenBank ID: AAY32927) is more similar to Sol i 4.01 (99.3%) than Sol g 4.01 (88%). Although *S. geminata* 4 venom proteins are found in tropical regions, different habitation sites have important effects, due to food, natural enemies and survival skills, which have led to various evolutionary adaptations [40].

### Expression and purification of the rSol g 4.1 protein

**Fig. 3:**
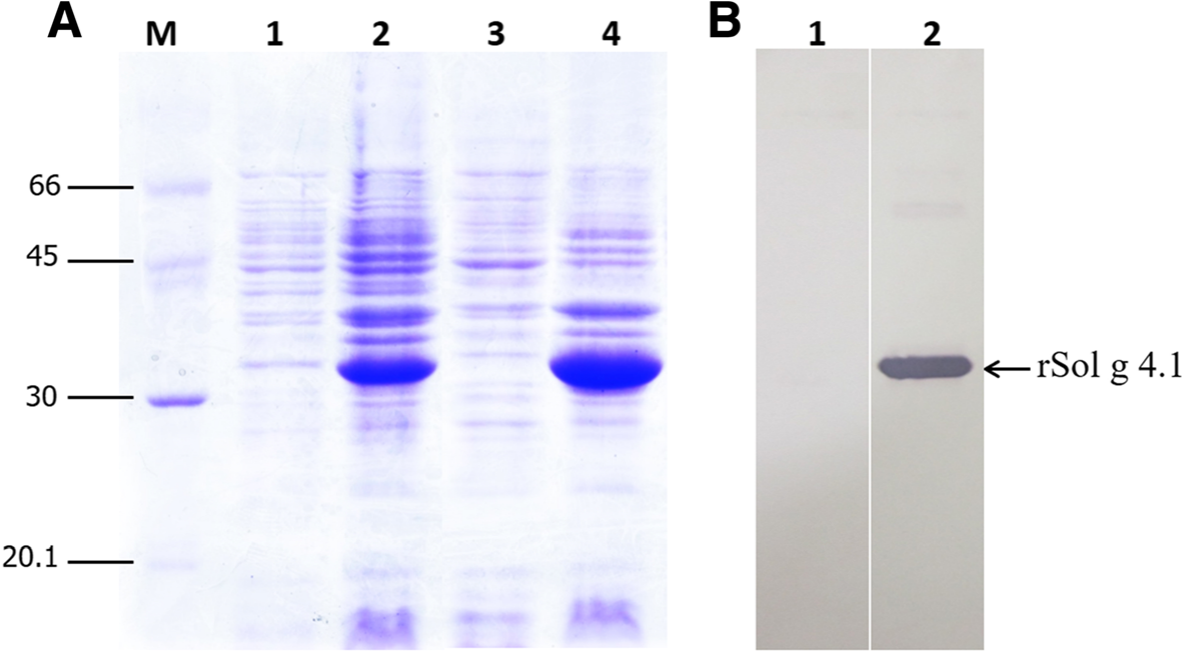
Determination of the overexpression of rSol g 4.1 protein by SDS-PAGE and Western blotting. **a** Protein expression patterns in BL21(DE3) pLysS competent cells cultured under optimal conditions obtained using SDS-PAGE. Lanes: M – molecular weight standards; 1 – expression without IPTG; 2 – culture grown in the presence of 0.4 mM IPTG for 8 h.; 3 – cell extract in solution; 4 – cell extract in pellet. **b** Western blot of the rSol g 4.1 protein using an anti-His tag antibody; lane 1 – cells lacking the rSol g 4.1 protein and lane 2 – expression of the rSol g 4.1 protein

**Fig. 4:**
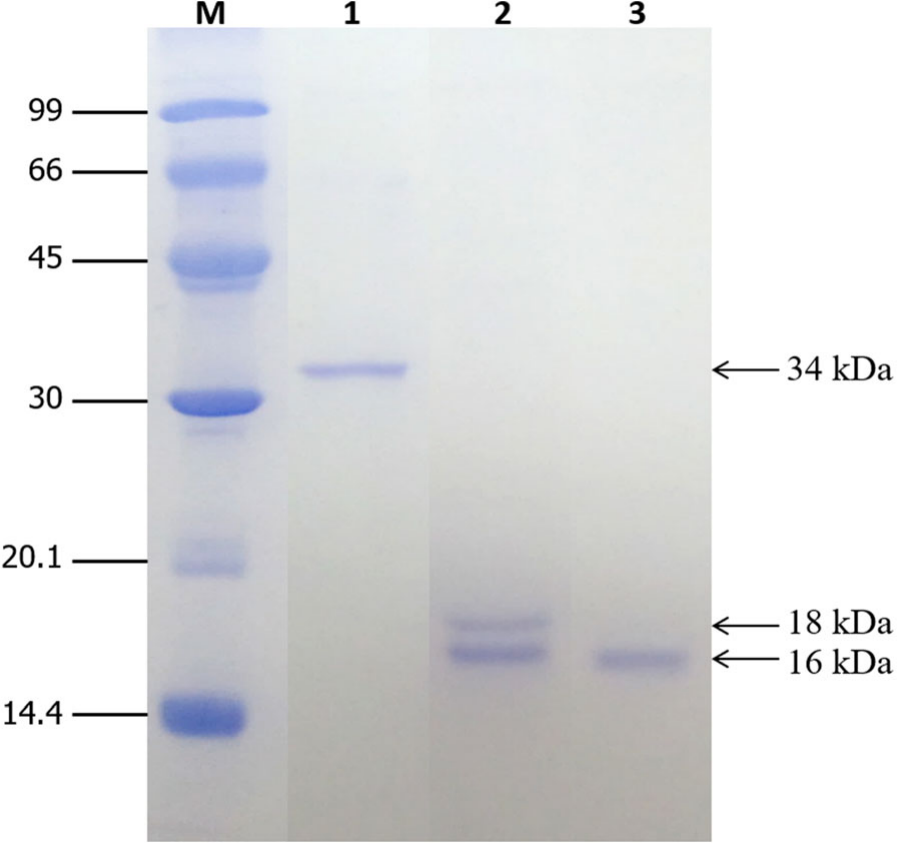
SDS-PAGE analysis of the purified rSol g 4.1 protein and product after cleavage of the N-terminal tag: lane M – molecular weight standards; lane 1 – purified rSol g 4.1 protein; lane 2 – cleavage of the tagged protein by one unit of enzyme for 7 h; and lane 3 – Sol g 4.1 protein after tag deletion and purification

The molecular weight of the expressed recombinant protein was approximately 34 kDa on SDS-PAGE. The expression levels of the recombinant clones were determined after an incubation with 0.2, 0.4, 0.6, 0.8 or 1.0 mM IPTG for 2, 4, 6, 8, and 10 h or overnight. The growth patterns were significantly different in terms of both IPTG concentration and induction times (data not shown). Therefore, the optimal conditions for culture growth were 0.4 mM IPTG and 8 h, as shown in Fig. 3a, lane 2. The rSol g 4.1 protein was expressed in inclusion bodies. Moreover, protein induction was confirmed by blotting lysates from induced and non-induced cultures with an anti-His tag antibody. The expressed protein strongly bound to antibodies, whereas proteins from non-induced culture did not bind (Fig. 3b). After purification, the rSol g 4.1 protein was dialyzed with a 12-kDa molecular weight cutoff membrane. The fusion protein was expressed as a monomer, and the purity was confirmed as a single band that represented 37% of the total proteins in Fig. 4, lane 1. The results for the cleavage of the tag from the rSol g 4.1 protein are not shown. The optimal conditions for the removal of the tag from the protein is one unit of enzyme and incubation for 7 h (Fig. 4, lane 2). The rSol g 4.1 protein was separated using a His GraviTrap column and analyzed on 13% SDS-PAGE gels, as shown in Fig. 4, lane 3. The purified protein represented approximately 2% of the total fusion protein.

LC-MS/MS was performed to ascertain the definite rSol g 4.1 protein. A single band representing the purified protein was trypsinized and subsequently identified using LC-MS/MS. The peptides were identified with the protein MASCOT search engine using the NCBI protein and SwissProt databases. A similarity search using FASTA revealed a very high homology to *S. geminata* venom allergen Sol g 4 for both the native and purified proteins, with molecular weights of 16,056 and 16,066 Da, respectively, as shown in Table 2, which also corresponds to the experimental weight of 16 kDa from Sol g 4, *S. geminata* venom, as described in our previous reports [26]. The software predicted that the Sol g 4.1 protein was a member of the allergenic protein family. Representative partial amino acid sequences of the Sol g 4.1 protein from the fragment spectra of the unassignable peptides in the tryptic digest were a 100% match after alignment and are shown in the yellow shaded region in Fig. 1.

**Table 2:** Protein identification of Sol g 4.1 from *S. geminata* venom

Spot	Matched protein	Accession no.	Score XC^a^	Theoretical MW/pI^b^	Peptide sequences^c^	Sequence coverage (%)	Species
Native band	Venom allergen 4	gi|14423995	109	16,056/9.87	K.GVLELTVYGK.K K.KGVLELTVYGK.K	9	*Solenopsis geminata*
Purified single band	Venom allergen Sol g 4.02	gi|7638030	368	16,066/9.87	K.GVLELTVYGK.K K.KGVLELTVYGK.K K.VYDRPGPIIER.S K.KVYDRPGPIIER.S K.CIKTVP.K	25	*Solenopsis geminata*

^a^Score XC obtained after LC-MS/MS analysis

^b^Theoretical molecular weight (MW) obtained after the LC-MS/MS analysis. The pI values were calculated using the ExPASy Peptide Mass program (http://web.expasy.org/compute_pi/)

^c^Deduced peptide sequence obtained after LC-MS/MS (the number of matching peptides is indicated in parentheses)

### Secondary structure analysis

The structure of the rSol g 4.1 protein lacking the tag consisted of 41.3% α-helices and 13.8% β-sheets after refolding, and unidentified structures constituted approximately 21.8% of the protein (Additional file 2). The denatured protein only exhibited 16.5% α-helices and 10.2% β-sheets; unidentified structures comprised 48.5% of the structure. Moreover, the secondary structure of the rSol g 4.1 protein showed 37% similarity to the *S. invicta* 2 monomer (Additional file 3), as predicted from schematic diagrams (PDBsum), which exhibits seven helices from the N-terminus to C-terminus in the overall structure. Thus, the refolded rSol g 4.1 protein likely adopts the native structure.

### Three-dimensional modeling of the predicted structure of the sol g 4.1 protein

The Sol i 2 (PDB code: 2ygu) chain A with a 2.60 Å resolution was used as a template; its X-ray structure is composed of two identical monomers [39]. The template showed the highest identity/similarity (35.90% with an E value of 1.0e^− 26^) to the Sol g 4.1 sequence. They are found in the same *Solenopsis* species venom. The Ramachandran plot displays the psi and phi backbone conformation angles for each amino acid residue in the Sol g 4.1 protein as shown in Additional file 4. The plot statistics for the model displayed residues falling in 95% of most favored regions, 4% of additional allowed regions, 0% of generously allowed regions and 1% of disallowed regions. Overall plot showed over 90% of residues within the most favorable region. Therefore, Sol g 4.1 model was an acceptable good quality model and can be used for further analysis.

**Fig. 5:**
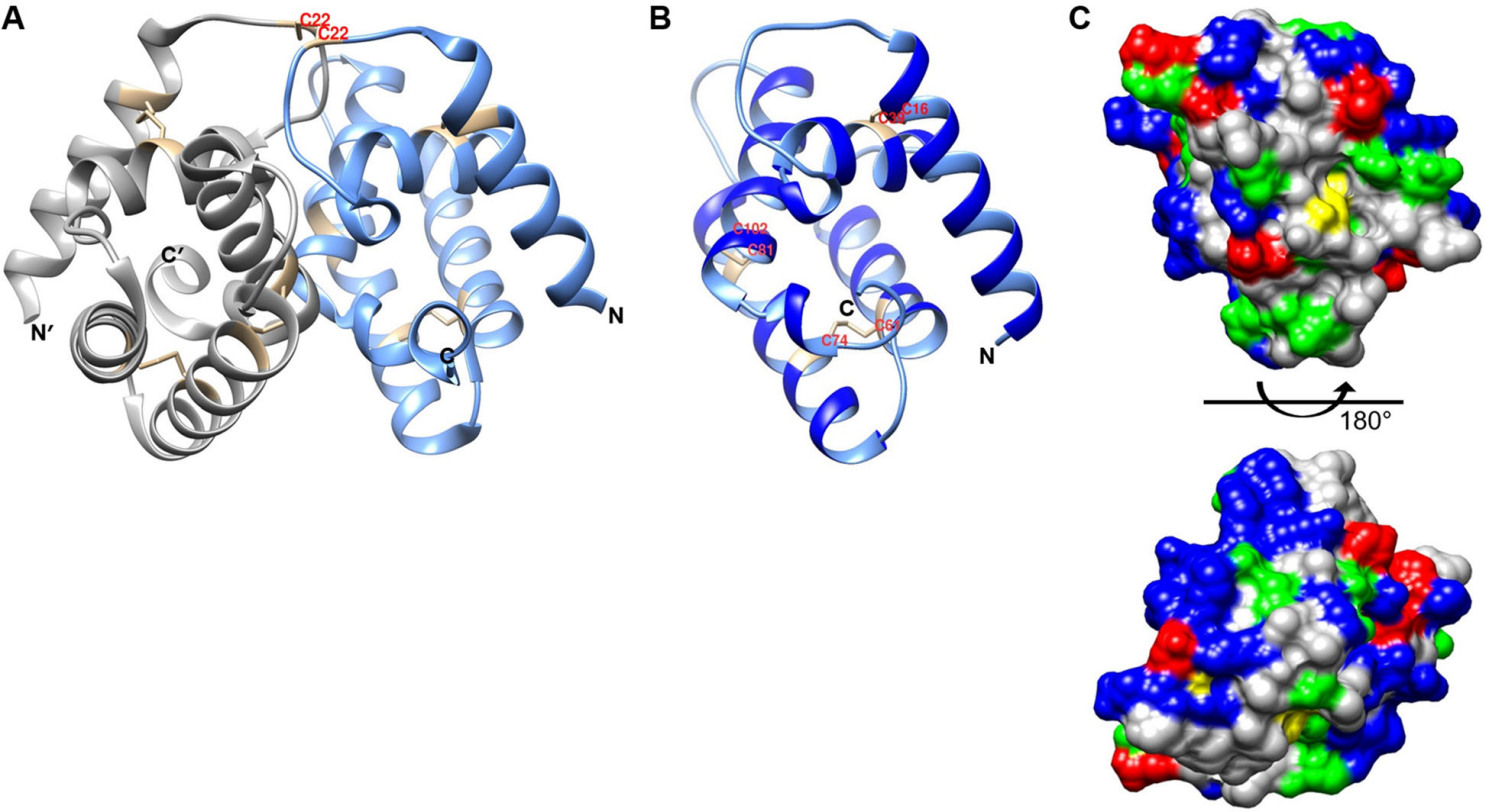
A ribbon diagram of the three-dimensional model of the predicted structure of the Sol g 4.1 protein constructed using *S. invicta* venom allergen Sol i 2 dimer (PDB accession no. 2ygu) as a template. The disulfide bonds are highlighted in tan. **a** Structural features of the Sol i 2 template dimerized by a disulfide bond on symmetrical residues Cys22. **b** The three-dimensional homology model of the predicted structure of the Sol g 4.1 protein revealed a structure stabilized by three disulfide bonds, and the molecular view is the same as the view shown for the right molecule in A. **c** The surface of Sol g 4.1 is marked according to amino acid residue properties: red – acidic residues; blue – basic residues; gray – apolar residues; green – polar residues; and yellow – aromatic residues. The molecule in the top view is the same as the molecule shown in B and has been rotated by 180° along the horizontal axis to show the bottom view. The model was obtained using Swiss-Model and was visualized with UCSF Chimera

Moreover, G-factor value from PROCHECK tool used for evaluation probability of all dihedral angles showed 0.14. Based on the model, the Sol g 4.1 protein consists of three disulfide bonds, which were predicted to stabilize structures (Cys16-Cys39, Cys61-Cys74 and Cys81-Cys102), and seven α-helices, which presumably surround the interior hydrophobic region. The comparison of the structures of the Sol g 4.1 protein and the template revealed that the Sol g 4.1 protein is present as a monomer, and the overall structure seems similar to part of the venom allergen 2 molecule (Fig. 5a and b). As reported in the study by Borer et al. [39], two alkanes (decane and undecane) and one alkene (β-farnesene), which are similar to alkane and alkene chains in these compounds, are attached to the sixth position of piperidine alkaloids and can bind to the hydrophobic pocket of Sol i 2. Thus, Sol i 2 is also conceivably involved in the transport of alkaloid derivatives from the site of synthesis to the venom reservoir or in the formation of a protective complex with the alkaloid in the venom duct.

A comparison of the amino acid residues in the three-dimensional models of the Sol g 4.1 and Sol i 2 structures showed that the interior face of the hydrophobic region is lined with 17 apolar residues and three polar residues (Fig. 2). Moreover, the structure of the Sol g 4.1 protein surface contains an unusually high number of charged residues that are evenly distributed on the surface, as shown in Fig. 5c. Overall, 35% of all residues on the surface of the Sol g 4.1 protein are charged: Asp, Glu, Lys, and Arg.

### Determination of allergenic properties

**Fig. 6:**
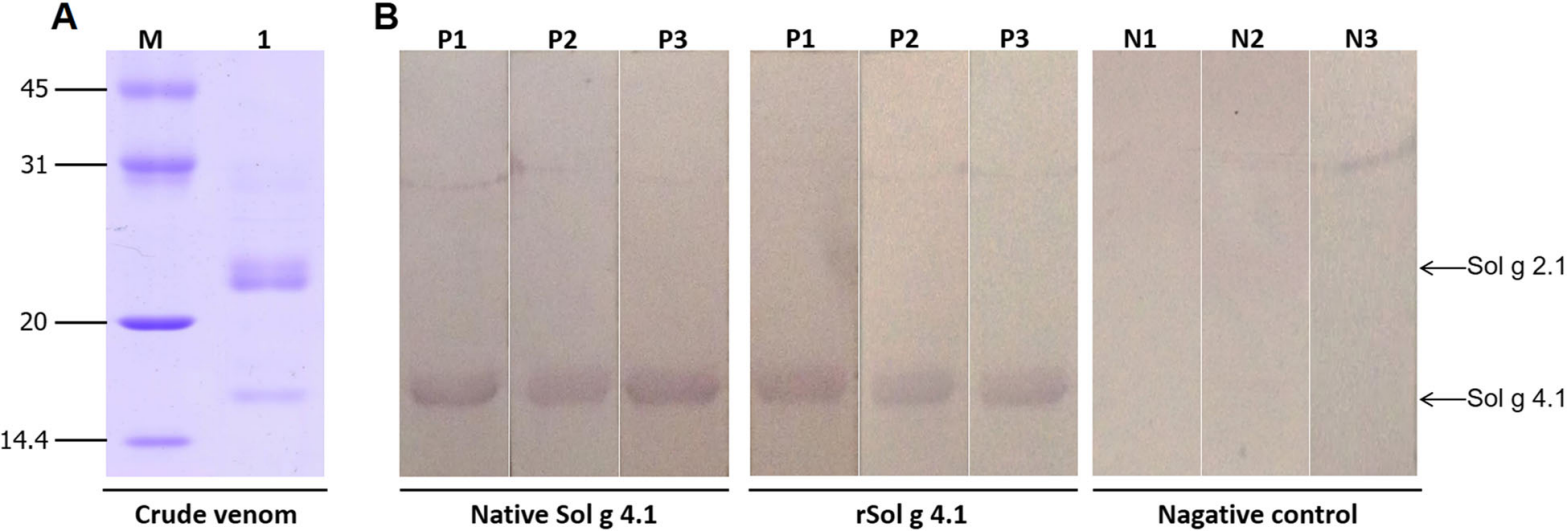
Allergenic analysis of native and recombinant Sol g 4.1 with anti-Sol g 4.1 IgE antibody. **a** Crude venom expression pattern, as determined by SDS-PAGE. **b** Determination of the allergenic properties of the Sol g 4.1 protein by producing an antiserum in mice and analyzing the product using Western blotting. Recognition of native Sol g 4.1 and rSol g 4.1 proteins by serum IgE in Sol g 4.1 protein-sensitized mice. Serum samples: P1-P3 = individual sera of Sol g 4.1 protein-sensitized mice; N1-N3 = serum from mice injected with PBS, acrylamide gel and adjuvant, respectively, as controls

An antiserum was produced in mice to determine the antigenic properties of the Sol g 4.1 protein. The Sol g 4.1 protein in crude venom was identified as a 16-kDa band on native PAGE gels in Fig. 6a, but the predicted molecular weight of its sequence is approximately 13,340 Da. Western immunoblotting analysis revealed a clear interaction between the produced antibody and both native and recombinant Sol g 4.1 proteins, which were approximately 16 kDa, while PBS, acrylamide gel and adjuvant controls did not produce bands, as shown in Fig. 6b. This result confirmed that we successfully produced a specific antibody in BALB/c mice (the anti-Sol g 4.1 IgE antibody) that recognized native and recombinant Sol g 4.1 proteins.

The antibody specifically recognized the Sol g 4.1 protein in its native form (Fig. 6b), suggesting that the antiserum did not show cross-reactivity to other proteins from crude venom. Interestingly, although Sol g 4.1 protein sequences share 42% identity with Sol g 2.1 sequences (unpublished data), they do not show immunological cross-reactivity, consistent with the results reported by Hoffman [1] for Sol i 2 and Sol i 4, which exhibit 35% sequence homology and no antibody cross-reactivity.

### Reduction of PD_50_ activity by the addition of rSol g 4.1

**Fig. 7:**
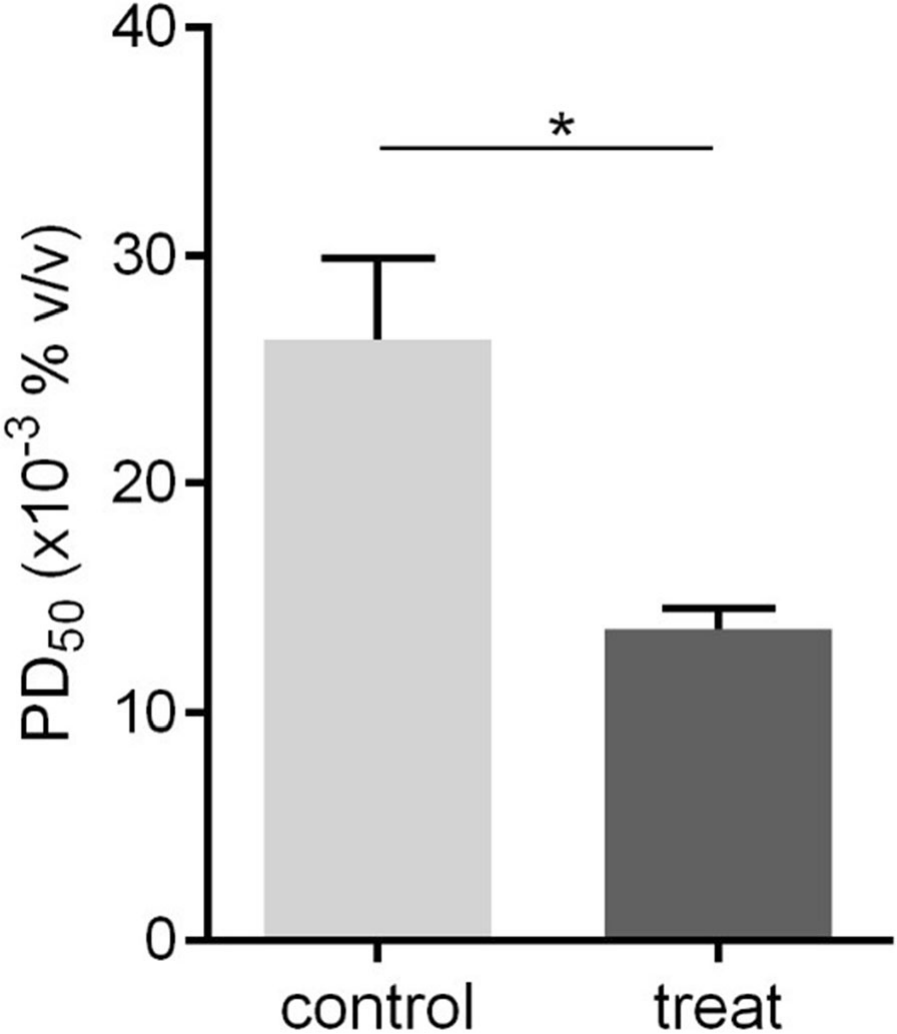
PD_50_ values for crickets injected with piperidine alone (control) and piperidine plus the rSol g 4.1 protein (treatment). The graph shows the means ± SEM for different percent dilutions of piperidine in the PD_50_ assay. *Values were significantly different from the control at *p* < 0.05

The PD_50_ was assayed in crickets in vivo to determine whether the refolded rSol g 4.1 protein lacking the tag altered the effects of piperidine alkaloids. The abdomen of the crickets was injected with PBS as a mock control or with a mixture of piperidine and protein and then incubated for 30 min. The PD_50_ of crude venom (positive control) in paralyzed crickets was 89 μg/g body weight, as described in our previous report [26]. First, the PD_50_ of piperidine in paralyzed crickets was approximately 0.027% (*v*/v) and designated PD_50_P1. Second, the injection of the rSol g 4.1 protein in crickets showed that the optimal concentration was 1.0 μg of protein (2.86 μg/g body weight), but the recombinant protein did not induce cricket paralysis. Finally, 1.0 μg of rSol g 4.1 protein was mixed with various concentrations of piperidine, and the PD_50_ value was determined to be approximately 0.013% (v/v) and designated PD_50_P2. Therefore, the rSol g 4.1 protein led to a significant decrease in PD_50_P1 to PD_50_P2, from 0.027 to 0.013% (*p* < 0.05), as shown in Fig. 7. The major chemical component of fire ant venom is piperidine alkaloids [41]. Piperidine derivatives are the main active components that paralyze prey [42]. Based on the results of these experiments, the Sol g 4.1 protein has important synergistic effect with the piperidine derivatives in venom.

## Discussion

The complete primary structure of the Sol g 4.1 protein was obtained in this study and showed high homology to Solenopsis 2 and 4 venom proteins, suggesting that they may perform similar functions and exhibit similar localization patterns. Based on three-dimensional model structures, Sol g 4.1 was identical to a portion of the Sol i 2 molecule. According to Borer et al. [39], the overall crystal structure of Sol i 2 is stabilized by three intramolecular disulfide bonds and one intermolecular disulfide bond, which differed from the Sol g 4.1 protein (only contains six cysteines), creating a hydrophobic pocket. Thus, the Sol g 4.1 protein is present as a monomer and that its structure is stabilized by three disulfide bonds. In addition, the Sol g 4.1 protein exhibited 21% identity to the hydrophobic ligand-binding proteins from the pheromone-binding protein/odorant-binding protein (PBP/OBP) family, which is normally composed of proteins with molecular weights of 12–16 kDa. The amino acid sequences are extremely diverse but are all distinguished by a pattern of six cysteines that form three disulfide bonds. The three-dimensional structure contains a cluster of six or seven α-helices surrounding the hydrophobic pocket in which the hydrophobic ligand binds [43, 44].

Whole-body extracts contain not only venom components but also proteolytic enzymes and various other soluble insect proteins. These soluble proteins can react with IgE antibodies that have been induced by proteins from other species, and the proteolytic enzymes can destroy venom allergens. Moreover, venom contains a significant concentration of piperidine alkaloids, which are difficult to completely remove from the proteins [41]. Allergenic proteins are also extremely difficult to purify from each other if they have similar pI values [15]. The expression of recombinant proteins will overcome the problem of obtaining large quantities from natural materials. Therefore, the expression and purification of the rSol g 4.1 protein in the *E. coli* system is a good choice for heterologous expression of recombinant proteins due to its capacity to produce abundant recombinant protein and easy manipulation.

The Sol g 4.1 protein was cloned into a pET-32a(+) vector containing the thioredoxin (Trx) tag, which can catalyze the formation of disulfides and promote the solubility of the target protein in the cytoplasm of *E. coli* [45]. However, the rSol g 4.1 protein was expressed as an insoluble protein, which may be affected by numerous parameters, including temperature [46], and then rSol g 4.1 was refolded by dialysis and we investigated its secondary structure, which was primarily α-helices. Venom protein expression in *E. coli* will save research cost and time, while expression in baculovirus-infected insect cells requires further study. As Solenopsis 4 proteins do not have carbohydrate determinants (CCDs) [36, 37], this study selected a quick and cheap system to express large concentrations of the Sol g 4.1 venom protein, which can be applied to allergenic testing of these venom proteins and could reduce the cost of this operation.

Based on the analysis of the allergenic properties, BALB/c mice generated an antibody in response to protein exposure [30] that strongly bound to the native and recombinant Sol g 4.1 proteins, suggesting that, as expected, the Sol g 4.1 protein was immunogenic in mice. This experiment was also supported by the finding that the surface of the Sol g 4.1 protein is composed of 35% charged residues (Asp, Glu, Lys, and Arg), a percentage that is considerably higher than the average value (27%) for normal proteins [47]. Charged amino acids often display significant contributions to the free energy of binding in protein-protein interactions and/or antigen-antibody complexes. The importance of charged surface residues in IgE binding and the allergenicity of the dust mite allergen Blo t 5 and other major allergens has been confirmed in mutagenesis studies [48–50]. Moreover, the sequence of the Sol g 4.1 protein produced in *E. coli* is highly conserved and displays greater than 86% identity to Sol i 4.01/Sol i 4.02 proteins produced using the same protein expression system, as identified as allergic individuals [36]. However, the full characterization of antigen-antibody recognition sites will require the elucidation of the complex structure of Sol g 4.1 protein with its specific antibodies, as allergen epitopes are continuous or discontinuous [51].

Moreover, we studied PD_50_ values by mixing piperidine alkaloids with rSol g 4.1 to verify the hypothetical functions of the Sol g 4.1 protein based on protein sequences and structural similarity to Sol i 2. The rSol g 4.1 protein may be involved in interactions with hydrophobic ligands, consistent with the results of the study by Borer et al. [39], who analyzed the role of the hydrophobic pocket in the allergenic Sol i 2 protein. The highest binding affinity was observed for hydrophobic ligands such as pheromones, fatty acids, or short-lived hydrophobic primers [52, 53]. Consistent with these findings, Das et al. [54] showed that Sol i 4.02 has an interior binding pocket with a size of approximately 0.4 nm^3^, and the interior pockets of *S. geminata* venom proteins bind to the alkaloid solenopsin A. Therefore, the Sol g 4.1 protein is also conceivably involved in the interaction with hydrophobic ligands.

Further studies are required to produce large amounts of soluble protein, which will aid in the study of the function of these extremely potent allergens. An analysis of the clear functions of the Sol g 4.1 protein should be performed, particularly a study that investigates its interactions with alkaloids/ligands and their localization patterns.

## Conclusions

To date, little is known about the biological activities of allergenic proteins from fire ant venom, including *S. geminata* venom. In our study, we describe the identification, expression and characterization of rSol g 4.1. Initially, rSol g 4.1 was expressed in inclusion bodies, and the structure of the refolded rSol g 4.1 protein was likely the native form, mainly α-helices, as determined by a secondary structure analysis. Both native and recombinant Sol g 4.1 proteins have a molecular weight of 16 kDa, although the amino acid sequence predicted a molecular weight of 13,340 Da. The predicted three-dimensional model showed three disulfide bonds that stabilized its structure. Solenopsis 2 and 4 venom proteins are unique ant venom proteins, including other Hymenoptera venom proteins [15, 19]. Based on a statistical analysis of cricket paralysis, Sol g 4.1 resulted in a significant decrease in PD_50_ values. Thus, similar to Sol g 4.02 [54], Sol g 4.1 seems to function by binding to hydrophobic ligands, such as pheromones and alkaloids. Based on the results of the allergenic test presented here, the anti-Sol g 4.1 IgE antibody responses observed in mice suggest that Sol g 4.1 is an allergenic protein.

## Additional files

Additional file 1: Phylogenetic relationships between the amino acid sequences of the *Solenopsis* species groups 2 and 4 proteins. *Bombyx mori* PBP (GenBank ID: P34174) is an outgroup. The evolutionary tree was analyzed using the neighbor-joining method. The percentage of replicate trees in which the associated taxa clustered together in the 1000 bootstrap character replicates is indicated for groups that appeared in ≥50% of bootstrap trees. Horizontal line distances are proportional to calculated phylogenetic differences. (JPG 2218 kb)

Additional file 2: CD spectrum of the Sol g 4.1 protein produced under denatured and refolded conditions. (JPG 34 kb)

Additional file 3: The topology diagram of the Sol g 4.1 protein created using PDBsum software shows the relative locations of α-helices, which are presented as red cylinders. The small arrows indicate the directionality of the protein chain from the N-terminus to the C-terminus. Numbers within the secondary structural elements correspond to the residue number in the protein. (JPG 262 kb)

Additional file 4: Ramachandran plot analysis of Sol g 4.1 model. The color codes are: red – most favorable regions, yellow – allowed regions, pale yellow – generously allowed regions; and white – disallowed regions. (JPG 288 kb)

## Abbreviations

PBP: Pheromone-binding protein
PD_50_: 50% of paralytic dose
rSol g 4.1 protein: Recombinant Sol g 4.1 protein
*S.*: *Solenopsis* species
Sol g 4: *Solenopsis geminata* venom allergen number 4
Sol i 2: *Solenopsis invicta* venom allergen number 2
Sol i 4: *Solenopsis invicta* venom allergen number 4
