# Supplementary figures and images for: Identification, expression and characterization of the recombinant Sol g 4.1 protein from the venom of the tropical fire ant Solenopsis geminata
Source: J Venom Anim Toxins Incl Trop Dis. 2018 Aug 29;24:23. doi: 10.1186/s40409-018-0159-6 (PMC6116302; doi:10.1186/s40409-018-0159-6)

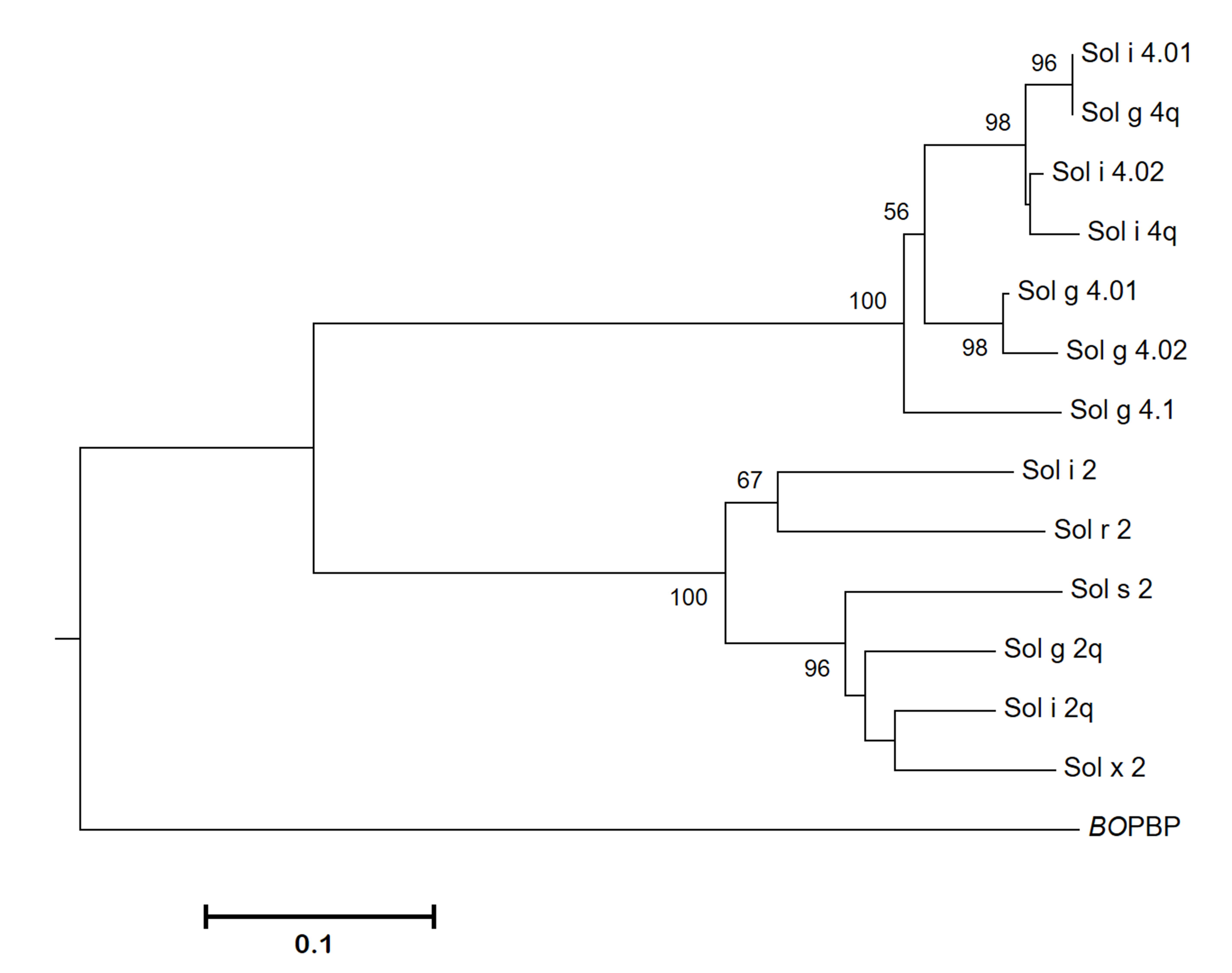

Supplement: Supplementary file 1 — Phylogenetic relationships between the amino acid sequences of the Solenopsis species groups 2 and 4 proteins. Bombyx mori PBP (GenBank ID: P34174) is an outgroup. The evolutionary tree was analyzed using the neighbor-joining method. The percentage of replicate trees in which the associated taxa clustered together in the 1000 bootstrap character replicates is indicated for groups that appeared in ≥50% of bootstrap trees. Horizontal line distances are proportional to calculated phylogenetic differences. (JPG 2218 kb) [file 40409_2018_159_MOESM1_ESM.jpg]

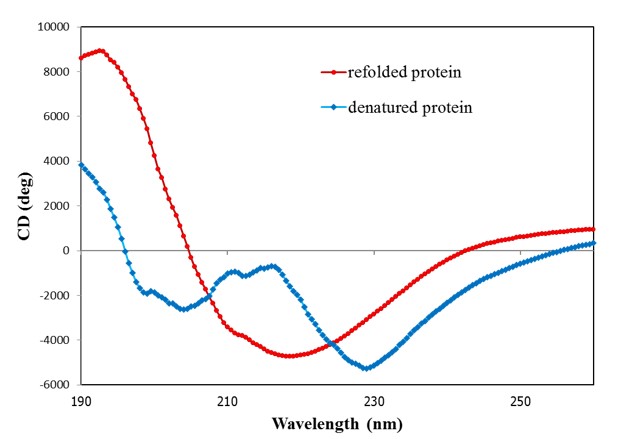

Supplement: Supplementary file 2 — CD spectrum of the Sol g 4.1 protein produced under denatured and refolded conditions. (JPG 34 kb) [file 40409_2018_159_MOESM2_ESM.jpg]

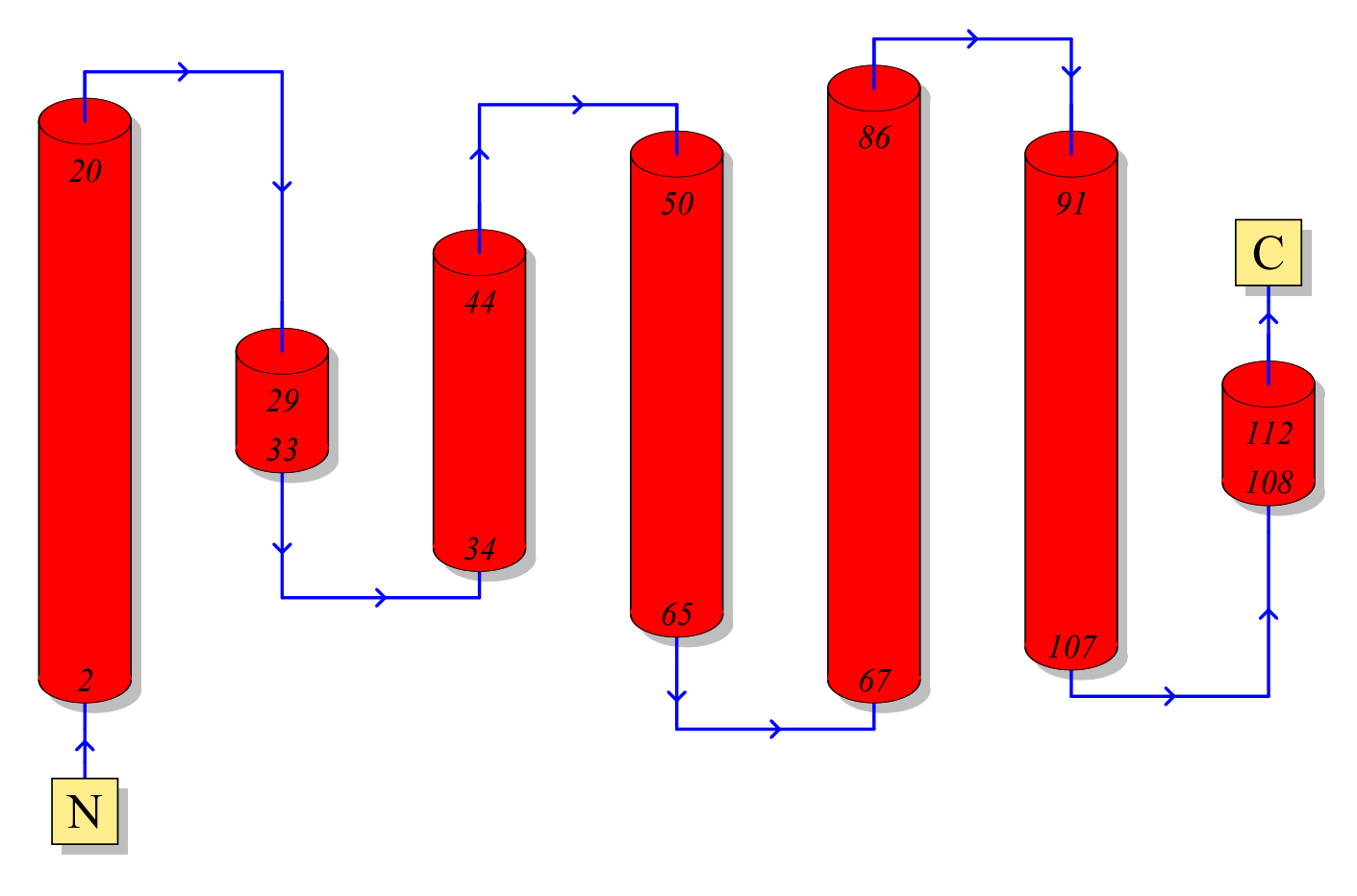

Supplement: Supplementary file 3 — The topology diagram of the Sol g 4.1 protein created using PDBsum software shows the relative locations of α-helices, which are presented as red cylinders. The small arrows indicate the directionality of the protein chain from the N-terminus to the C-terminus. Numbers within the secondary structural elements correspond to the residue number in the protein. (JPG 262 kb) [file 40409_2018_159_MOESM3_ESM.jpg]

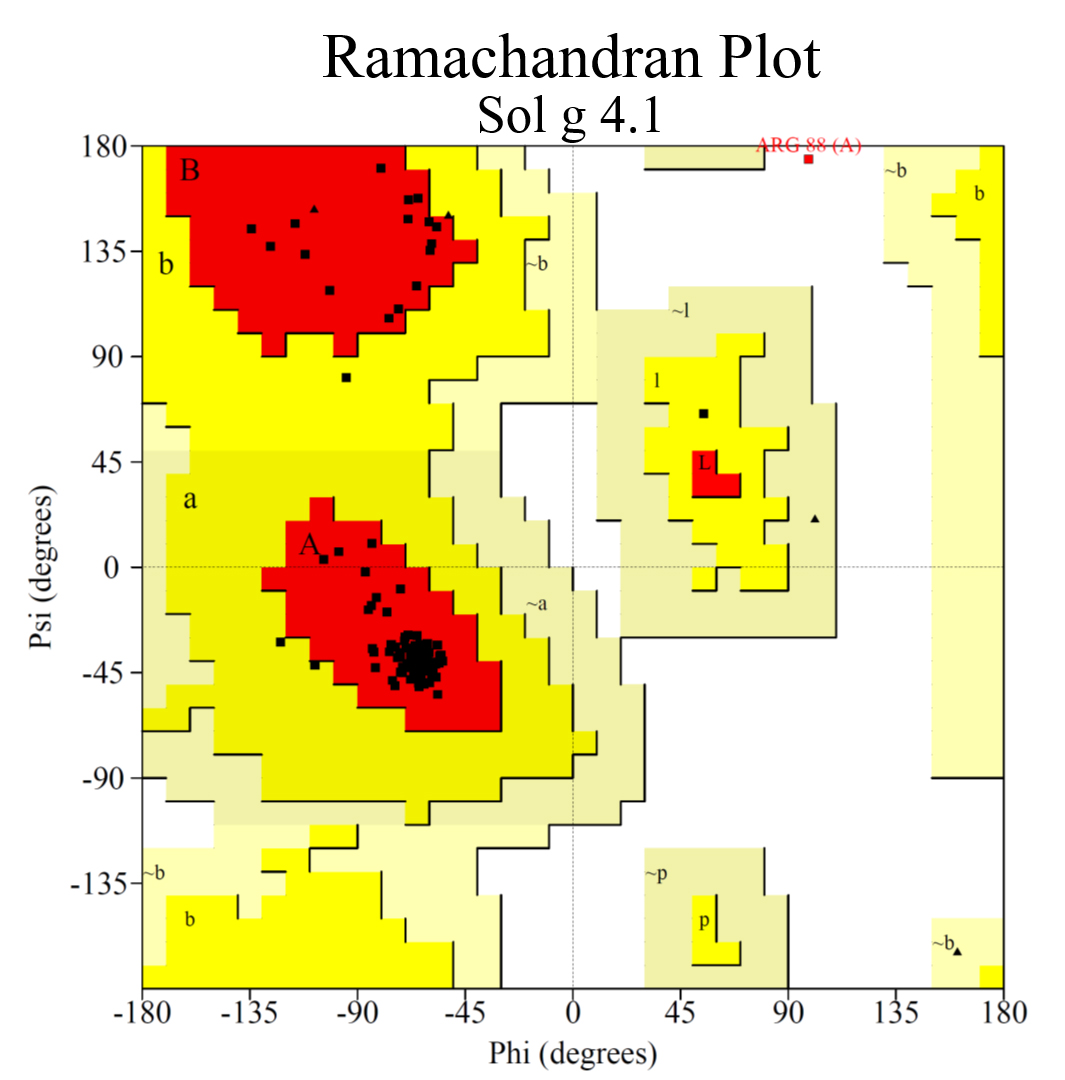

Supplement: Supplementary file 4 — Ramachandran plot analysis of Sol g 4.1 model. The color codes are: red – most favorable regions, yellow – allowed regions, pale yellow – generously allowed regions; and white – disallowed regions. (JPG 288 kb) [file 40409_2018_159_MOESM4_ESM.jpg]
